# NADPH oxidase mediates the oxygen-glucose deprivation/reperfusion-induced increase in the tyrosine phosphorylation of the *N*-methyl-D-aspartate receptor NR2A subunit in retinoic acid differentiated SH-SY5Y Cells

**DOI:** 10.1186/1750-2187-7-15

**Published:** 2012-09-08

**Authors:** Phillip H Beske, Darrell A Jackson

**Affiliations:** 1 From the Department of Biomedical and Pharmaceutical Sciences and the Center for Structural and Functional Neuroscience, The University of Montana, Missoula, MT, 59812, USA; 2 Department of Biomedical and Pharmaceutical Sciences, College of Health Professions and Biomedical Science, Skaggs Building 394, The University of Montana, Missoula, MT, 59812, USA

**Keywords:** NMDA receptor, Oxygen-glucose deprivation/Reperfusion, NADPH oxidase, Oxidative stress, Src Family kinases, Excitotoxicity

## Abstract

**Background:**

Evidence exists that oxidative stress promotes the tyrosine phosphorylation of *N*-methyl-D-aspartate receptor (NMDAR) subunits during post-ischemic reperfusion of brain tissue. Increased tyrosine phosphorylation of NMDAR NR2A subunits has been reported to potentiate receptor function and exacerbate NMDAR-induced excitotoxicity. Though the effect of ischemia on tyrosine phosphorylation of NMDAR subunits has been well documented, the oxidative stress signaling cascades mediating the enhanced tyrosine phosphorylation of NR2A subunits remain unclear.

**Results:**

We report that the reactive oxygen species (ROS) generator NADPH oxidase mediates an oxidative stress-signaling cascade involved in the increased tyrosine phosphorylation of the NR2A subunit in post-ischemic differentiated SH-SY5Y neuroblastoma cells. Inhibition of NADPH oxidase attenuated the increased tyrosine phosphorylation of the NMDAR NR2A subunit, while inhibition of ROS production from mitochondrial or xanthine oxidase sources failed to dampen the post-ischemic increase in tyrosine phosphorylation of the NR2A subunit. Additionally, inhibition of NADPH oxidase blunted the interaction of activated Src Family Kinases (SFKs) with PSD-95 induced by ischemia/reperfusion. Lastly, inhibition of NADPH oxidase also markedly reduced cell death in post-ischemic SH-SY5Y cells stimulated by NMDA.

**Conclusions:**

These data indicate that NADPH oxidase has a key role in facilitating NMDAR NR2A tyrosine phosphorylation via SFK activation during post-ischemic reperfusion.

## Background

*N*-methyl *D*-aspartate receptor (NMDAR) activation is one of the many important steps necessary to elicit long-term potentiation (LTP). This is accomplished by NMDARs not only through calcium entry, but also through the initiation of several downstream effectors. NMDAR activation has been shown to lead to the activation of NADPH oxidase, resulting in superoxide production [1]. The generation of superoxide anions in the central nervous system (CNS) plays an intriguing dual role, delicately balancing normal function and the potential to cause cellular damage. Under normal physiologic conditions, superoxide has been shown to be necessary for LTP in the hippocampus [2]. Recent studies have demonstrated that upon NMDAR activation, a resulting superoxide burst arises from NADPH oxidase [1,3], which when blocked with pharmacological inhibition of NADPH oxidase consequently prevents LTP in the hippocampus [4]. Spatial localization of NADPH oxidase subunits revealed immunohistochemical staining of a membrane bound subunit of NADPH oxidase, gp91^phox^, as well as the cytosolic components of NADPH oxidase, p67^phox^ and p47^phox^*,* in the soma and dendrites of mouse hippocampal slices [5]. This localization is consistent with previous studies that have demonstrated a similar spatial expression of NMDARs [6]. Since superoxide is short lived due to its highly reactive properties, its generation needs to be in close proximity to its protein targets to serve as a possible signaling molecule. Given the proximity of NADPH oxidase to the synapse, and the localized production of superoxide it produces, the enzyme can thus serve as a signaling hub to affect post-synaptic receptor function.

However, while ROS may be necessary for the processes of LTP, its un-controlled overproduction leads the cell into a state of oxidative stress. Abramov *et al.*[7] demonstrated that superoxide anions are generated in a tri-phasic manner in neurons following 40 minutes of oxygen-glucose deprivation/reperfusion (OGD/R). The three superoxide bursts were identified sequentially as originating from mitochondrial, xanthine oxidase, and NADPH oxidase sources through the use of pharmacological inhibition as well as knock out studies, with the tertiary superoxide burst arising from NADPH oxidase occurring during reperfusion. Since NADPH oxidase reduces molecular oxygen to superoxide, the lack of available oxygen during OGD limits the reaction. However, upon re-introduction of oxygen, a rapid and sustained burst of superoxide from NADPH oxidase occurs. Furthermore, this superoxide burst is involved in the processes of cellular death, as pharmacological inhibition of NADPH oxidase during ischemia/reperfusion (I/R) with the NADPH oxidase inhibitor apocynin is highly neuroprotective [8].

NMDARs play a key role in the post-ischemic mechanisms of excitotoxic neuronal injury and death. The massive increase in extracellular glutamate known to occur during I/R injury leads to an over-stimulation of NMDARs, a key factor in neuronal death which results from increased calcium overload and pathological ROS production. In addition to excessive glutamate stimulation, NMDARs also undergo phosphorylation mediated conformational changes following I/R that potentiates the effect of glutamate NMDAR stimulation. In particular, a rapid and sustained increase in tyrosine phosphorylation of the NMDAR NR2A subunit has been reported following I/R [9-13]. Increased tyrosine phosphorylation of the NR2A subunit functionally potentiates NMDAR currents by increasing the likelihood of the receptor being in an open conformation, and decreases the probability of the receptor being in an inactive state [14].

In recent years, evidence has documented the role of Src Family Kinases (SFKs) in up-regulating the activity of NMDARs via tyrosine phosphorylation [15] of residues located on the C-terminus of NR2 subunits [16]. Through inhibition of SFK activity, phosphorylation of NR2 subunits is prevented and the elicitation of LTP is blocked [15]. While this process is important for synaptic plasticity, an enhancement of the effect of glutamate during an excitotoxic event only exacerbates calcium cytotoxic damage. SFKs have been demonstrated to modulate NMDAR function following I/R [12]. Direct inhibition of SFKs with PP2 was shown to provide neuroprotection following ischemic insult [12,17]. Additionally, the interaction of SFKs with postsynaptic density protein 95 (PSD-95), a scaffolding protein that concentrates NMDARs at the post-synaptic density, is an important mechanism underlying I/R-induced increases in the tyrosine phosphorylation of NR2A and NR2B subunits. Suppression of PSD-95 expression diminishes tyrosine phosphorylation of both subunits following I/R [18]. Furthermore, inhibiting Src interaction with PSD-95 prevents the I/R-induced increase in tyrosine phosphorylation of the NMDAR NR2A subunit [18]. Taken together, the process of SFKs interacting with PSD-95 facilitates the tyrosine phosphorylation of NMDAR NR2 subunits, thereby enhancing calcium entry through the receptor.

While SFKs have been reported to increase in activity following I/R [17,12], the upstream signals leading to its activation remain largely unresolved. SFKs have been reported to be redox sensitive in nature [19,20], although the ROS source responsible for its redox-induced activation during I/R remains unknown. In this study using retinoic acid differentiated SH-SY5Y neuroblastoma cells, a model known to be susceptible to NADPH oxidase-induced cell death [21], we sought to determine whether superoxide production from NADPH oxidase was involved in the activation of SFKs associated with PSD-95, thereby mediating the tyrosine phosphorylation of the NMDAR NR2A subunit.

## Results

### Increased NADPH oxidase activity following oxygen glucose deprivation in retinoic acid differentiated SH-SY5Y cells

**Figure 1:**
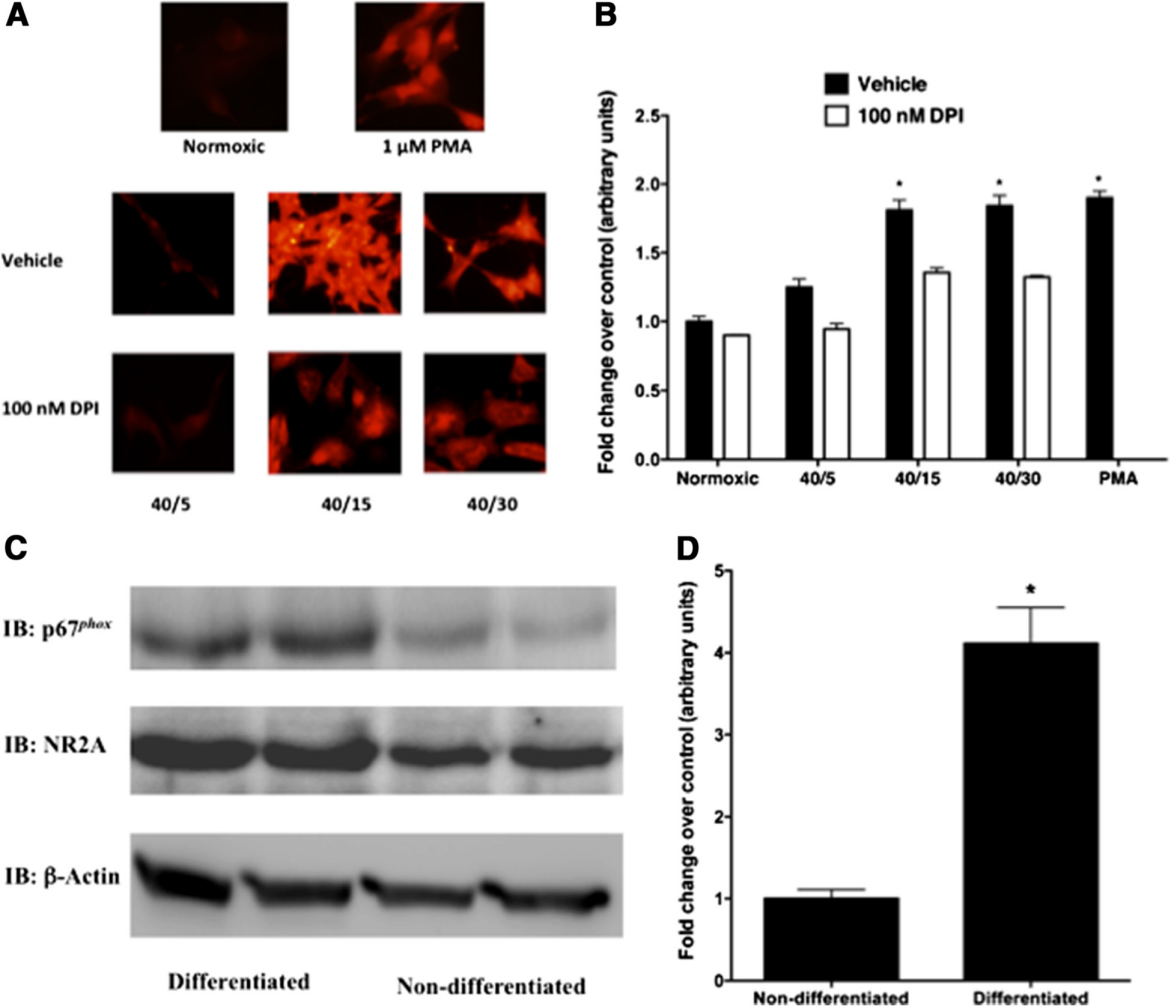
**Increased ROS generation during reperfusion of OGD subjected cultures of retinoic acid differentiated SH-SY5Y involves NADPH oxidase activity.** Representative fluorescent images from three independent experiments of reactive oxygen species production following OGD (**A**) with vehicle (1:1000 DMSO) or DPI (100 nM) detected using dihydroethidium (DHE). Spectrophotometric quantification of reactive oxygen species following OGD (**B**) with vehicle (1:1000 DMSO) or DPI (100 nM) utilizing nitrobluetetrazolium chloride (NBT). (**C**) Western blot shown is representative of three independent experiments comparing p67^phox^, NR2A subunit protein expression in non- and differentiated SH-SY5Y cells. (**D**) Quantification of the relative densitometry of p67^phox^ immunoreactive band in non- and differentiated SH-SY5Y cells. Data represent fold change of OGD/R groups versus normoxic control group (arbitrary units) ± S.E.M from three separate experiments that consisted of at least 6 determinents (askteriks * indicates a p <0.05 from vehicle treated normoxic control; ANOVA with *post hoc* Bonferroni test). Phorbol 12-myristate 13-acetate (PMA; 1 μM for 15 minutes) was used as a positive control for NADPH oxidase activity in both the DHE and NBT assays. Quantitative data from p67^phox^ protein expression represent fold change of differentiated SH-SY5Y compared to non-differentiated SH-SY5Y cells (arbitrary units) ± S.E.M from three separate experiments (askteriks * indicates a p <0.01 from non-differentiated control; student *t*-Test).

To examine whether SH-SY5Y cells served as an appropriate cell model to study the effects of NADPH oxidase activity on NMDAR NR2A subunits following OGD/R, we examined the expression of NADPH oxidase cytosolic subunit p67^phox^ in retinoic differentiated and non-retinoic acid differentiated SH-SY5Y cells. Consistent with previous findings [21], protein expression of the NADPH oxidase cellular component p67^phox^ in retinoic acid differentiated SH-SY5Y increased 4-fold as compared to non-differentiated SH-SY5Y cells (Figure 1C and D). NR2A protein expression was also increased in retinoic acid treated SH-SY5Y cells (Figure 1C). Retinoic-acid differentiated SH-SY5Y cells were reported to be more susceptible to oxidative stress-induced cellular death than non-differentiated SH-SY5Y cells [21]. Therefore, differentiated SH-SY5Y cells were used throughout this study to examine whether NADPH oxidase was involved in the OGD/R-induced increase in tyrosine phosphorylation of the NR2A subunit. To determine whether activation of NADPH oxidase activity occurred during OGD incubation or during the subsequent reperfusion, as previously reported in cortical neuronal cells [7], we subjected 6-day retinoic acid differentiated SH-SY5Y neuroblastoma cells to 40-minutes of OGD followed by reperfusion of cultures for various incubation times in the presence of dihydroethidium (DHE) (Figure 1A). Reperfusion of OGD cultures lead to superoxide generation that was maximal between 15- and 30-minutes (Figure 1A and B). Treatment of cultures with the NADPH oxidase inhibitor DPI (100 nM) during reperfusion drastically decreased DHE fluorescence, suggesting that NADPH oxidase may be responsible for superoxide production during reperfusion of OGD subjected cultures. Treatment of cultures with 1 μM phorbol 12-myristate 13-acetate (PMA) for 15 minutes, a known positive control for NADPH oxidase activity [22], yielded a strong fluorescent signal. As anticipated based upon Western blot results showing a low level of p67^phox^ expression, an increase in ROS production during reperfusion following 40 minutes of OGD in non-differentiated SH-SY5Y cells was not observed (Figure 1C and D). We also observed an increase in the reduced product of nitroblue tetrazolium chloride (NBT) in differentiated SH-SY5Y cultures subjected to OGD/R (Figure 1B), which was similar to the time course to ROS production observed with DHE. Treatment of OGD cultures with DPI during reperfusion blunted NBT reduction (Figure 1B), suggesting that NADPH oxidase activation underlies the ROS production during reperfusion of OGD cultures.

### OGD/Reperfusion-induced Increased tyrosine phosphorylation of NMDAR NR2A subunit

**Figure 2:**
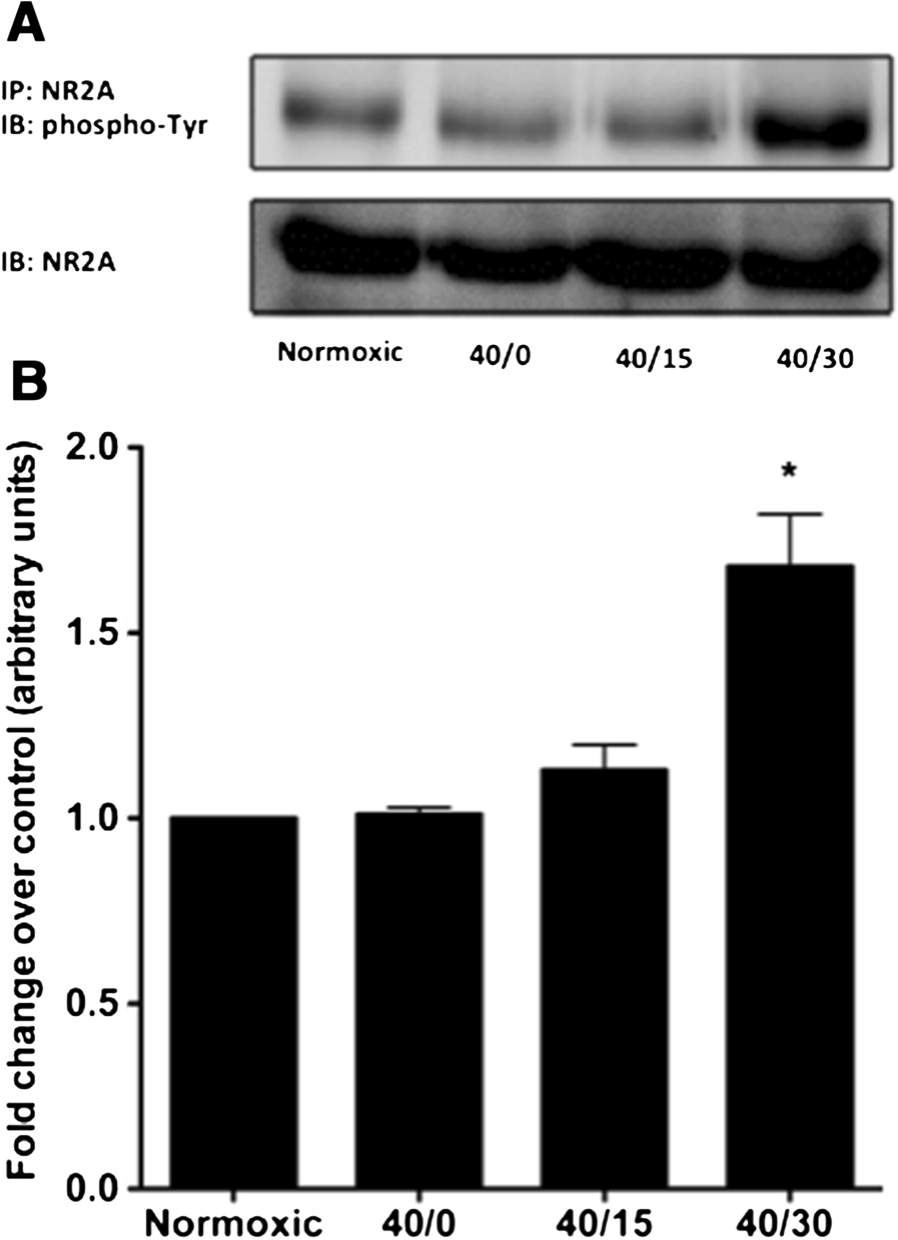
**OGD/R promotes the increase in tyrosine phosphorylation of the NMDAR NR2A subunit in differentiated SH-SY5Y cells.** Immunoprecipitation and immunoblotting of lysates prepared from differentiated SH-SY5Y cells subjected to OGD/R reveals a significant increase in tyrosine phosphorylation of the NR2A subunit. (**A**) Western blot shown is representative of three independent experiments. (**B**) Quantification of the relative densitometry of the phospho-Tyr-NR2A immunoreactive band. Data represent fold change of OGD/R groups compared to normoxic control group (arbitrary units) ± S.E.M from three separate experiments. (askteriks * indicates a p <0.01 from normoxic control; ANOVA with *post hoc* Bonferroni test).

Previous studies have demonstrated that there is a rapid and sustained increase in tyrosine phosphorylation of the NMDAR NR2A subunit during post-ischemic reperfusion [9]. Therefore, experiments were performed to examine whether OGD or subsequent reperfusion of OGD subjected cultures would lead to an increase in tyrosine phosphorylation of the NMDAR NR2A subunit. In non-differentiated SH-SY5Y cells subjected to OGD/R, there was no observable increase in tyrosine phosphorylation of the NR2A subunit as compared to time-matched normoxic control (data not shown). In contrast, we found a significant increase in tyrosine phosphorylation of the NR2A subunit at 30-minutes of reperfusion in differentiated SH-SY5Y cells subjected to OGD incubation (Figure 2A and B). This increase in tyrosine phosphorylation coincided with the time course in which we observed maximal ROS generation (Figure 1B). Collectively, these results indicate that NMDAR NR2A subunit undergoes an increase in tyrosine phosphorylation during reperfusion of OGD subjected differentiated SH-SY5Y cells. This increase in tyrosine phosphorylation of the NR2A subunit coincided with increased ROS production.

### Inhibition of NADPH oxidase attenuates OGD/Reperfusion-induced increase in NR2A tyrosine phosphorylation

**Figure 3:**
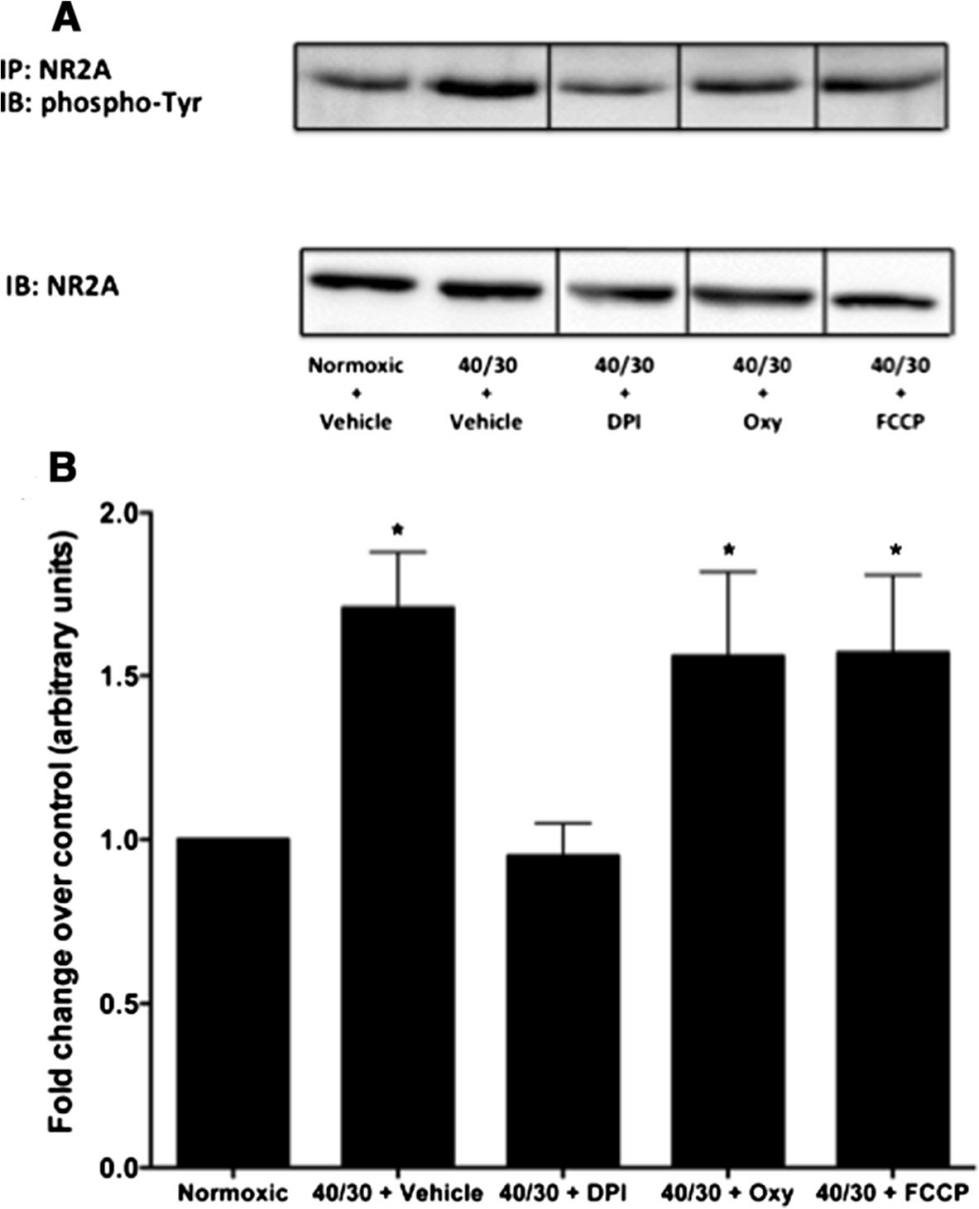
**Inhibition of NADPH Oxidase activity blocks the OGD/R-induced increase in tyrosine phosphorylation of the NMDAR NR2A.** Inhibition of superoxide generation from xanthine oxidase with oxypurinol (1 μM) and mitochondria with FCCP (0.5 μM) failed to prevent the OGD/R-induced increase in tyrosine phosphorylation of the NMDAR NR2A subunit. However, inhibition of NADPH Oxidase with DPI (100 nM) was found to block the increase in tyrosine phosphorylation of the NR2A subunit. Representative Western blot (**A**) of three independent experiments of phospho-Tyr-NR2A with vehicle (1:1000 DMSO), DPI, oxypurinol, and FCCP. (**B**) Quantification of the density of the phospho-Tyr-NR2A band. Data represent fold change of OGD/R treated groups compared to normoxic control (arbitrary units) ± S.E.M from three separate experiments. (askteriks * indicates a p <0.05 from normoxic control; ANOVA with *post hoc* Bonferroni test).

To examine whether mitochondrial, xanthine oxidase, or NADPH oxidase is the ROS generator that mediates the oxidative stress signaling cascade responsible for the increase in tyrosine phosphorylation of the NR2A subunit in differentiated SH-SY5Y cells, cultures were treated during reperfusion with 0.5 μM of the mitochondrial depolarization uncoupler carbonyl cyanide 4-(trifluoromethoxy) phenylhydrazone (FCCP), the xanthine oxidase inhibitor oxypurinol (1 μM), or the NADPH oxidase inhibitor DPI (100 nM). Previous data from this study had shown a decreased ROS production with concentrations of DPI as low as 100 nM, while Abramov *et al.*[7] had previously demonstrated inhibition of xanthine oxidase and mitochondrial ROS production with the concentrations indicated. Treatment of cultures with FCCP or oxypurinol failed to prevent increase in tyrosine phosphorylation of the NR2A subunit during 30-minutes of reperfusion of OGD subjected SH-SY5Y cultures (Figure 3B). However, inhibition of NADPH oxidase activity with DPI prevented the increase in tyrosine phosphorylation of the NR2A subunit at 30 minutes of reperfusion following OGD. There were no changes in total protein levels of NR2A in cultures treated with DPI, oxypurinol, or FCCP. These results indicate that NADPH oxidase underlies the oxidative stress-signaling cascade responsible for the OGD/R-induced increase in NR2A tyrosine phosphorylation.

### OGD/Reperfusion-induced association between Src-family kinase and PSD-95 is prevented with inhibition of NADPH oxidase activity

**Figure 4:**
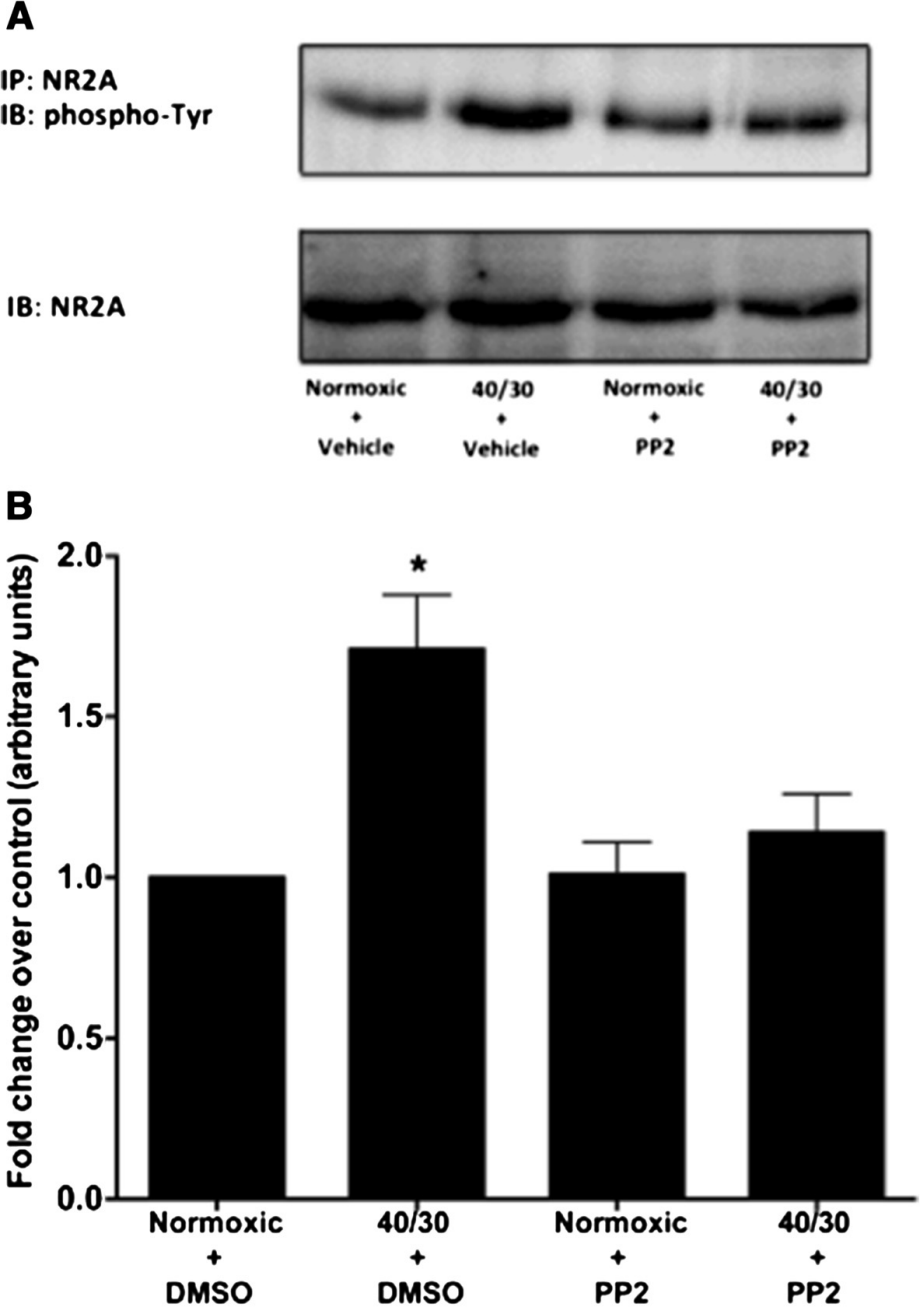
**The OGD/R-induced increase in tyrosine phosphorylation of the NMDAR NR2A subunit is attenuated by treatment with the Src Inhibitor PP2.** Inhibition of Src Family Kinase activity with the selective inhibitor PP2 rescued the increase in tyrosine phosphorylation of the NMDAR NR2A subunit. (**A**) Representative Western blot from 3 independent experiments illustrates the dampened tyrosine phosphorylation of NR2A with direct inhibition of Src Family Kinases during reperfusion following OGD with PP2 (1 μM). (**B**) Quantification of the density of the phospho-Tyr-NR2A band. Data represent fold change of treated groups compared to normoxic vehicle treated group (arbitrary units) ± S.E.M from three separate experiments. (askteriks * indicates a p <0.05 from normoxic control; ANOVA with *post hoc* Bonferroni test).

**Figure 5:**
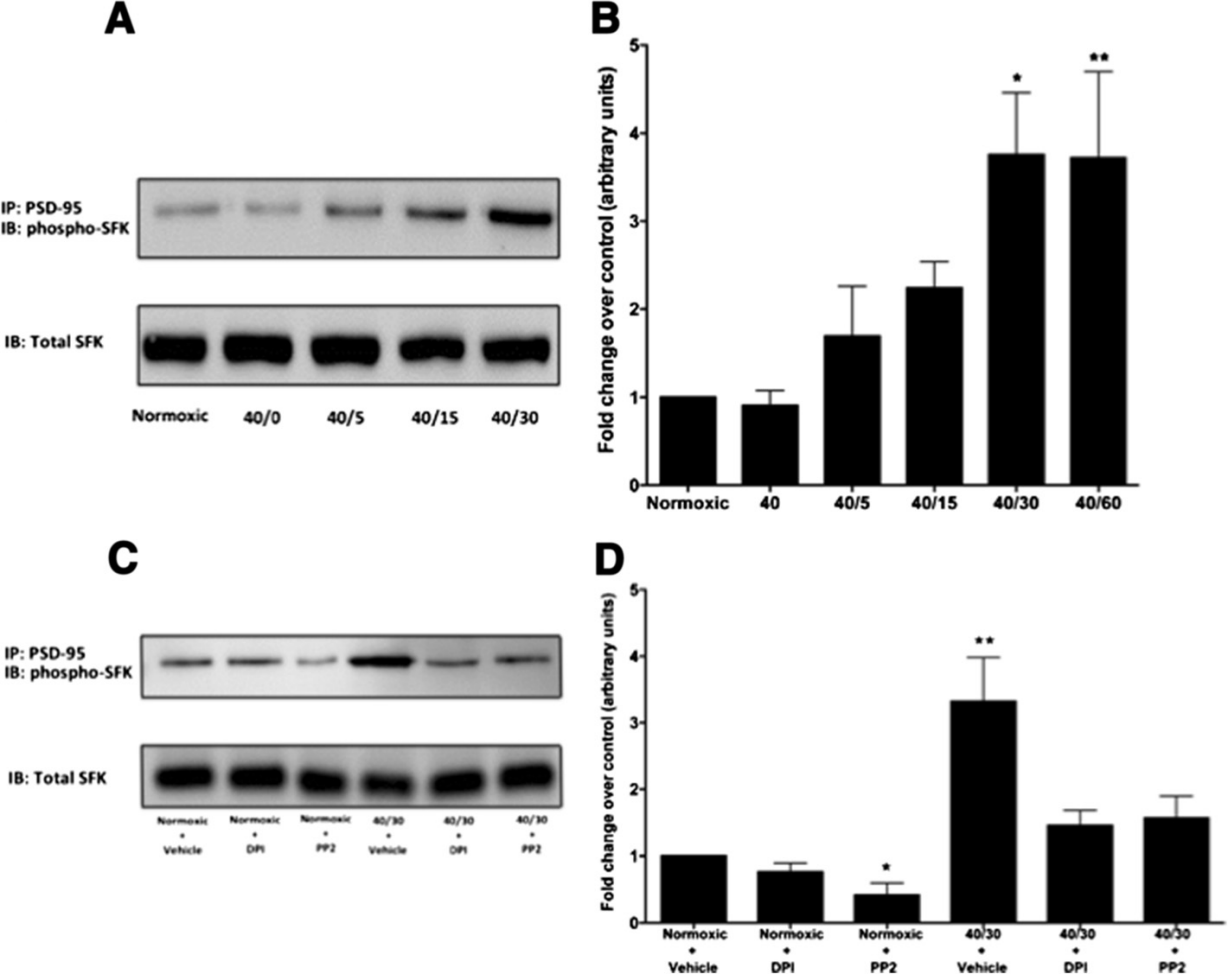
**OGD/R promotes the interaction between activated Src Family Kinases and PSD-95, which is reduced with NADPH oxidase inhibition differentiated SH-SY5Y cells.** (**A**) Immunoprecipitation of PSD-95 and immunoblotting with phospho-SFK(Tyr416) shows the increase in the active form (phospho-Tyr416) of SFKs bound to PSD-95 with treatment of OGD/R compared to normoxic controls (Norm). Western blot is representative of three independent experiments. (**B**) Quantification of the density of the phospho-SFK(Tyr416) band after PSD-95 immunoprecipitation. (**C**) Representative Western blot from immunopreciptation of PSD-95 from three independent experiments illustrates the dampening of activated SFKs bound to PSD-95 with DPI (100 nM) or PP2 (1 μM) treatment during reperfusion following 40 minutes of OGD. (**D**) Quantification of the density of the phospho-SFK(Tyr416) band after PSD-95 immunoprecipitation. (B) Data represent fold change of OGD/R groups compared to normoxic control (arbitrary units) ± S.E.M from three separate experiments. (D) Data represents fold change of treated groups compared to vehicle treated normoxic control group (arbitrary units) ± S.E.M from three separate experiments. (askteriks * indicates a p <0.05 from normoxic control; ** indicates a p <0.001; ANOVA with *post hoc* Bonferroni test).

SFKs have been shown to mediate increases in tyrosine phosphorylation of NMDAR subunits after transient cerebral ischemia [9-12,17]. To determine whether SFKs were involved in mediating OGD/R-induced increase in tyrosine phosphorylation of the NR2A subunit in differentiated SH-SY5Y cells, cultures were treated with the potent SFK inhibitor PP2. Administration of PP2 (1 μM) immediately following OGD blunted the increase in tyrosine phosphorylation of the NR2A subunit (Figure 4B). In a previous study, it was reported that suppression of PSD-95 expression reduced the increase in tyrosine phosphorylation of the NR2A subunit after transient brain ischemia in the hippocampus [18]. Therefore, to determine whether activation of NADPH oxidase facilitated the interaction of activated SFKs associating with the scaffolding postsynaptic density protein 95 (PSD-95), we performed an immunoprecipitation of PSD-95 combined with immunoblotting for activated SFKs. We found that the active form of SFKs (phospho-Tyr416) associated with PSD-95 during reperfusion of OGD cultures (Figure 5A), as early as 5 minutes of reperfusion and sustained for at least 30 minutes of reperfusion. Inhibition of NADPH oxidase significantly reduced the association of activated SFKs with PSD-95 during reperfusion of OGD cultures (Figure 5C). These results indicate that the OGD/R-induced activation of NADPH oxidase facilitates the interaction of activated SFKs with PSD-95 thereby facilitating the tyrosine phosphorylation of the NR2A subunit.

### Inhibition of NADPH oxidase following OGD/Reperfusion dampens the exacerbated effect of NMDA induced cellular death

**Figure 6:**
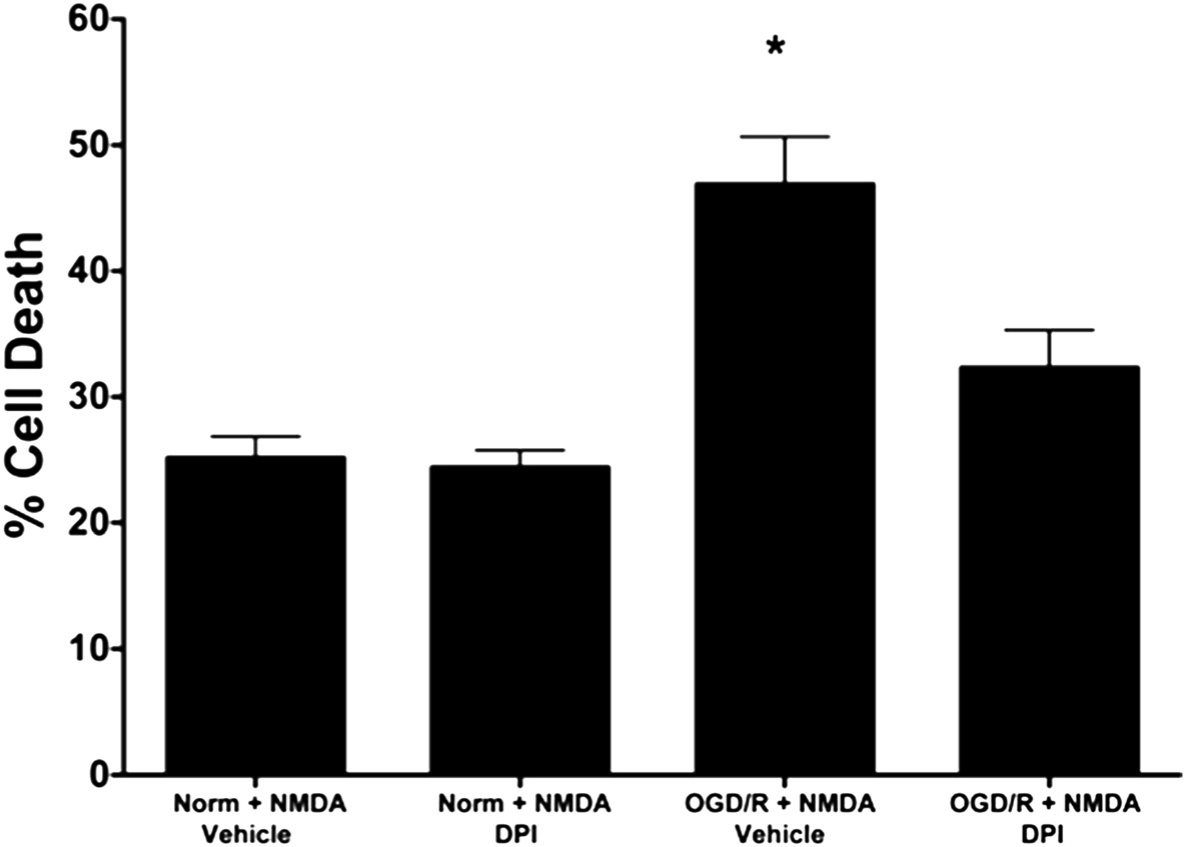
**Inhibition of NADPH oxidase activity decreases NMDAR-mediated cell death following exposure to OGD.** Differentiated SH-SY5Y cells exhibit an increased susceptibility to cell death after 6 hours of NMDA (5 mM) stimulation following 40 minutes of OGD as assessed by the ethidium homodimer exclusion assay. This enhanced NMDAR-induced cell death is diminished with NADPH oxidase inhibition using DPI (100 nM). Data represent % mean cell death ± S.E.M from three independent experiments consisting of 10–12 determinants (askteriks * indicates a p <0.05 from normoxic control; ANOVA with *post hoc* Bonferroni test)

One of the functional consequences of NMDAR NR2A tyrosine phosphorylation is a potentiation of NMDAR currents [14]. Through an increased permeability to calcium, the effect of glutamate stimulation of NMDARs is amplified, thereby exacerbating calcium overload and subsequent cell death. Previous studies have demonstrated a marked decrease in cellular viability of SH-SY5Y cells when exposed to 0.25-5 mM NMDA stimulation [23]. To determine if the OGD/R-induced activation of NADPH oxidase contributed to NMDAR mediated cellular death, we used an ethidium homodimer exclusion assay as a marker for cellular viability. We found that following 40 minutes of OGD, treatment of differentiated SH-SY5Y cells with NMDA (5 mM) elicited an increased susceptibility to cellular death after 6 hours as compared to normoxic NMDA stimulated controls (Figure 6). However, inhibiting NADPH oxidase during the 6 hours of NMDA treatment with DPI (100 nM) following the 40 minutes of OGD, significantly reduced susceptibility to NMDA mediated cell death. These results indicate that the NMDAR mediated cell death in post-ischemic differentiated SH-SY5Y cells involves NADPH oxidase.

## Discussion

While numerous studies have identified changes in NMDAR subunit function following ischemia [11,12], as well as the downstream signaling cascades leading to cellular death [24,25], upstream mechanisms leading to these changes remain largely unresolved. Therefore, experiments were conducted to identify the source of ROS generation involved in the oxidative stress-signaling cascade responsible for the increase in tyrosine phosphorylation of the NMDAR NR2A subunit. Additionally, we performed experiments to establish the mechanism in which the OGD/R-induced activation of NADPH oxidase leads to this increase in tyrosine phosphorylation of the NR2A subunit, as well as to demonstrate a functional consequence of NADPH oxidase activity on enhanced NMDAR-induced cellular death following OGD/R.

Studies performed by Abramov *et al.*[7] demonstrated in neuronal cultures that both during OGD and the subsequent reperfusion, superoxide is generated in a tri-phasic manner from distinct sources. The primary and secondary burst arise from the mitochondria and the enzyme xanthine oxidase during the OGD phase. During the reperfusion following OGD, a tertiary burst of superoxide was observed [26] which was shown to be a result of NADPH oxidase activity. Further studies have also reported that NADPH oxidase activity results in the production of superoxide *in vivo* in the hippocampus of adult mice subjected to ischemia/reperfusion [26]. We therefore sought to determine the temporal pattern of ROS production following exposure to OGD/R in retinoic acid differentiated SH-SY5Y cells utilizing DHE fluorescence as well as NBT reduction. ROS production, while observed to occur during 40 minutes of OGD, was maximally increased by 15 minutes of reperfusion and was drastically blunted when NADPH oxidase was inhibited with DPI, both in the DHE and NBT assays (Figure 1A and B). While superoxide production from NADPH oxidase has been shown to contribute to neuronal death [8,26] following stroke, its basal activity under physiologic conditions is thought to be critical in the processes of LTP as demonstrated by an inhibition of LTP in knock-out studies of mice lacking a functional NADPH oxidase holoenzyme [4]. Therefore, under pathologic conditions such as ischemia/reperfusion, we sought to determine if superoxide produced from NADPH oxidase played a role in mediating the increased tyrosine phosphorylation of the NMDAR NR2A subunit following OGD/R.

Modifications on the C-terminal regions of NMDAR subunits in the brain via phosphorylation are thought to play a key role in neuronal development, synaptic plasticity, and a variety of pathologic conditions [27]. While increases in both serine and threonine phosphorylation does occur on NR1 and NR2 subunits, potentiation of NMDA currents seems to be accomplished via direct tyrosine phosphorylation of NR2 subunits by protein tyrosine kinases [16].

Tyrosine phosphorylation of the NR2A increases the probability that the receptor will enter a long-lived open conformation, as well as decrease the likelihood of the receptor entering a long-lasting closed state [14]. This increase in tyrosine phosphorylation ultimately affects the amount of calcium that is able to enter through the receptor, resulting in an increased effect of glutamate upon NMDAR stimulation. We found that a significant increase in tyrosine phosphorylation of the NMDAR NR2A subunit occurred during reperfusion of OGD subjected in retinoic acid differentiated SH-SY5Y cells. As indicated previously, ROS generation by NADPH oxidase occurs during post-ischemic reperfusion [7]. While numerous reports have established that ischemic insult results in an increase of NMDAR tyrosine phosphorylation [9,10], the upstream signaling pathways leading to this increase in phosphorylation have not been fully described. We found that inhibition of NADPH oxidase activity with DPI significantly attenuated the OGD/R-induced increase in NR2A tyrosine phosphorylation. Inhibition of mitochondrial ROS production with FCCP or xanthine oxidase ROS production with oxypurinol had no significant effect on reducing NR2A tyrosine phosphorylation, suggesting that the key superoxide source for signaling for changes in NMDAR NR2A tyrosine phosphorylation is NADPH oxidase. These findings are consistent with previous studies [13,28], as inhibition of NADPH oxidase with mGluR1 antagonism reduced the increase in tyrosine phosphorylation of the NR2A subunit following I/R, ultimately decreasing infarct size *in vivo*. However, these studies did not report underlying mechanisms between NADPH oxidase activation and an increase in NMDAR NR2 subunit phosphorylation status.

SFKs, originally discovered as a proto-ocongene yet later found to be highly expressed in fully differentiated mature neurons [29], are important in mediating NMDAR tyrosine phosphorylation [11,14]. More specifically, SFKs are activated following ischemia and form interactions with NMDARs [12]. We demonstrated that OGD/R-induced increase tyrosine phosphorylation of the NR2A subunit involves the activity of SFKs by dampening the increase with SFK inhibition (Figure 4). This SFK mediated tyrosine phosphorylation of the NR2A subunit appears to also involve interaction with PSD-95. This is consistent with the findings of Hou *et al.*[18] that reported I/R-induced increase in NR2 subunit phosphorylation requires activated Src to bind to PSD-95 prior to phosphorylating NR2 subunits. We have extended this finding by demonstrating that inhibition of NADPH oxidase leads to a diminished SFK interaction with PSD-95. Additionally, the OGD/R-induced increase in tyrosine phosphorylation of the NMDAR NR2A subunit was diminished through NADPH oxidase inhibition. Therefore, this result suggests that OGD/R-induced increase in NR2A tyrosine phosphorylation involves the NADPH oxidase mediated SFK interaction with PSD-95. SFKs have been shown to play a role in mediating cellular death during stroke, as treatment with the inhibitor PP2 has been demonstrated to be neuroprotective [12,17]. In retinoic acid differentiated SH-SY5Y cells, we demonstrated that treatment with PP2 significantly reduced the OGD/R-induced tyrosine phosphorylation of the NR2A subunit, further indicating the importance of SFKs in mediating the tyrosine phosphorylation of NMDARs. A possible explanation of SFK activation during OGD/R lies in the redox sensitive nature of SFK activity [19], which would explain the large increase in SFK activation observed when in a state of oxidative stress such as stroke.

Lastly, we sought to determine if inhibition of NADPH oxidase could protect against the exacerbated excitotoxic effect of NMDA stimulation following OGD/R. Wang *et al.*[8] has previously showed the neuroprotective effect of NADPH oxidase inhibition *in vivo* following I/R. However, the mechanism providing this neuroprotection was not fully investigated. Physiologic LTP studies have demonstrated that pharmacologic inhibition of NADPH oxidase diminishes the ability of receptor signaling to potentiate synaptic currents [4]. While necessary for LTP under physiologic conditions, the dampening of excitatory receptor signaling could be beneficial in pathologic conditions leading to calcium overload via excitotoxicity as observed during stroke. Through inhibition of NADPH oxidase activity with DPI, enhanced cell death after NMDA stimulation following OGD/R was significantly rescued. A plausible mechanism for such protection could be explained by the prevention of the increase in tyrosine phosphorylation of the NMDAR NR2A subunit with NADPH oxidase inhibition, thereby diminishing the enhanced excitotoxic effect of NMDAR stimulation. The focus of this study was specifically aimed at elucidating the signaling mechanism involved in OGD/R-induced increase in NMDAR NR2A tyrosine phosphorylation. I/R-induced SFK-mediated increases in NMDAR NR2B subunit tyrosine phosphorylation have also been reported [9,12], but further studies need be performed to investigate a possible role of NADPH oxidase in signaling for these events following I/R.

We have demonstrated that NADPH oxidase is activated and is a major source of ROS generation during reperfusion following OGD in retinoic acid differentiated SH-SY5Y cells. This ROS burst from NADPH oxidase is important in mediating the activation of SFKs and its interaction with PSD-95, which consequently are responsible for the observed increase in the tyrosine phosphorylation of the NMDAR NR2A subunit. Collectively, this data indicates an upstream mechanism leading to changes in the phosphorylation of NMDAR NR2A subunit, thereby ultimately affecting the potentiated properties of the receptor in post-ischemic tissue.

## Conclusion

In summary, the data presented in this study demonstrate that OGD/R-induced Src activation and subsequent increase in tyrosine phosphorylation of the NMDAR subunit NR2A involves NADPH oxidase activation. Additionally, findings from this study suggest that NADPH oxidase activation underlies the oxidative stress signaling cascade responsible for the susceptibilities of neuronal cells to NMDAR mediated cellular death.

## Methods

### Materials

Ethidium homodimer was bought from Molecular Probes (Eugene, OR, USA). HALT protease inhibitor cocktail was bought from Pierce (Rockford, IL, USA). All other chemicals used in the study were purchased from Sigma (St. Louis, MO, USA).

### Cell Culture and differentiation

Human SH-SY5Y neuroblastoma cells were used throughout the study. SH-SY5Y cells were cultured in Dulbecco’s Modified Eagle’s Medium: Ham’s Nutrient Mixture F-12, 1:1 (D-MEM/F-12) purchased from ATCC (Manassas, VA, USA) supplemented with 10% fetal bovine serum (Gibco, Grand Island, NY, USA) and Penicillin (100 μg/mL) / Streptomycin (100 U/mL). For immunoprecipation and lysate preparation, SH-SY5Y cells were seeded onto 10 cm dishes at a density of approximately 4 x 10^6^ cells per dish. Approximately 24 hours later, cells were differentiated by treatment with complete D-MEM/F-12 supplemented with 10 μM retinoic acid for 6 days and fresh media containing retinoic acid was changed every 48 hours [21].

### Oxygen-Glucose Deprivation/Reperfusion

Anoxia was achieved by incubating the cultures in a controlled humidified hypoxic glove box (Coy Laboratories, Grass Lake, MI, USA) for 40 minutes at 37°C. The gas mixture in the incubator was 0% O_2_, 5% CO_2_, and 95% N_2_. Anoxia was verified using an oxygen meter with an O_2_ microelectrode (OM-4; Microelectrodes Inc., Bedford, MA, USA). Glucose free DMEM without serum was placed in the hypoxic glove box overnight at 0% O_2_ at 37°C to de-oxygenate the media. Prior to oxygen-glucose deprivation/reperfusion (OGD/R), cells were serum starved for 4 hours. Cells were then rinsed 3 times with phosphate buffered saline (PBS) prior to placement in the hypoxic glove box at 37°C. The de-oxygenated glucose and serum free media was then added to the dishes in the glove box and the cultures were incubated for 40 minutes. Following OGD, the anoxic glucose free media was removed and cultures were returned to a normoxic (ambient air O_2_ levels; 18-21% O_2_) tissue culture incubator with serum free media containing glucose (± drug treatment) for the variousincubation periods as described per individual experiments.

### Detection of Reactive Oxygen Species Generation

Dihydroethidium (DHE) experiments were modified and adapted from the method described by Abramov *et al.*[7]. Briefly, SH-SY5Y cells were plated at 1 x 10^5^ onto nitric acid washed glass coverslips in 35 mm dishes. Approximately 24 hours after seeding, cells were differentiated as previously described. OGD/R experiments were performed with 5 μM DHE present in the media throughout the entire experiment and no pre-loading period was performed. Following treatment, cells were washed 3 times with cold PBS (4°C, pH 7.4) and fixed with cold 4% paraformaldehyde/PBS (4°C, pH 7.4). Cells were visualized using an Olympus 1X71 inverted microscope equipped with a 60X oil immersion objective. Images were obtained with Olympus Image Manager. For the nitrobluetetrazolium (NBT) assay, methods were adapted from Aukrust *et al.*[30]. Briefly, 5 x 10^4^ cells were plated onto 12-wells plates and differentiated with retinoic acid as described above. After 4 hours of serum starvation, media containing 0.1;% (w/v) NBT was added to each well and incubated for 1 hour. After loading, excess NBT was washed away 3 times with warmed PBS (37°C, pH 7.4) and the OGD/R treatments were performed along with time-matched normoxic controls. Following treatments, cells were fixed with absolute methanol (−20°C) and washed twice with room temperature 70% methanol. Plates were allowed to air dry before dissolving the formazan deposits by the addition of 2 M KOH and 100% DMSO to each well. The absorbance was then measured at 630 nm with a Spectra Max Gemini M2 plate reader (Molecular Devices, Sunnyvale, CA, USA).

### Immunoprecipitation of NR2A and PSD-95

SH-SY5Y cells were plated onto 10 cm tissue culture dishes at a density of 4 x 10^6^ cells per dish as previously described. Approximately 24 hours later, cultures were differentiated with retinoic acid as previously described. Prior to treatment, the cultures were serum starved for 4 hours. Immediately following treatment, two 10 cm dishes of cells were washed twice with cold PBS (4°C, pH 7.4), and were pooled to ensure adequate protein yield after harvesting in HEPES lysis buffer (50 mM HEPES, pH 7.5, 0.5% NP-40, 250 mM NaCl, 2 mM EDTA, 10% Glycerol, 1 mM sodium orthovanadate, 1 mM sodium fluoride, 1 μg/mL benzamidine, 2 μl/mL Halt Protease Inhibitor Cocktail Kit). Cells were briefly sonicated for a 5 second burst at 25% power output with a VirTis Ultrasonic Cell Disrupter 100 (Gardiner, NY, USA). Cell lysates were spun at 1,000 x *g* to remove nuclei and cell debris. Protein concentration was determined using a bicinchoninic acid assay (BCA), and lysates (500 μg/sample) were then pre-cleared using Protein-A/G 50/50 mix of agarose beads for 1 hour at 4°C followed by incubation with primary antibody overnight (affinity-purified rabbit-polyclonal NR2A, Sigma, St. Louis, MO, USA or an affinity-purified rabbit-monoclonal PSD-95, Cell Signal, Beverley, MA, USA) at 4°C. The immunocomplex was then incubated for 4 hours with 50 μL Protein-A/G beads at 4°C with rotation before being washed 3 times with 500 μL of HEPES lysis buffer. Samples were eluted from the agarose beads by treatment with Laemmli buffer and heat (100°C) and then subjected to 7.5% sodium dodecyl sulfate polyacrylamide gel electrophoresis (SDS-PAGE). After transfer to nitrocellulose membranes (Bio-Rad, Berkeley, CA, USA), blots were blocked for 1 hour at room temperature with 5% BSA in Tris Buffered Saline, 0.1% Tween 20 (TBS-T) and incubated with an affinity-purifed phospho-tyrosine (1:2000, Sigma, St. Louis, MO, USA) or phospho-Src Family Kinase (Tyr416) (1:1000, Cell Signaling Technology, Beverly, MA, USA). Immunoreactive bands were then visualized and captured with a Fuji imaging system using enhanced chemiluminescence after adding HRP conjugated secondary antibodies (1:2000) of Goat anti-Mouse-HRP or Mouse anti-Rabbit-HRP (IgG Light chain specific) both purchased from Jackson ImmunoResearch (West Grove, PA, USA). Bands were analyzed using Image-Gauge software.

### Immunoblotting

Samples for direct immunoblotting were subjected to 7.5% SDS-PAGE, transferred to nitrocellulose membranes and blocked with 53% BSA/TBS-T as previously described. Blots were incubated with primary antibody overnight at 4°C at the concentration indicated. The affinity purified NR2A rabbit polyclonal antibody (1:2000) was purchased from Sigma (St. Louis, MO, USA). The affinity purified rabbit-polyclonal p67*phox* antibody (1:1000) was purchased from Millipore (Billerica, MA, USA). The affinity purified rabbit-monoclonal Src Family Kinase (1:1000) antibody was purchased for Cell Signaling Technology (Beverly, MA, USA). The affinity-purified mouse-monoclonal ß-actin (1:5000) antibody was purchased from CalBiochem/EMD (Darmstadt, Germany). Goat anti-Mouse-HRP and Goat-anti-Rabbit-HRP secondary antibodies (1:2000) were purchased from Jackson ImmunoResearch (West Grove, PA, USA). Immunoreactive bands were visualized and captured with a Fuji imaging system using enhanced chemiluminescence after adding HRP conjugated secondary antibodies. Bands were analyzed using Image-Gauge software.

### Quantification of cell death

Cell viability was determined using an ethidium homodimer exclusion test. Briefly, approximately 5 x 10^4^ cells were plated onto 12 well plates and 24 hours later cells were differentiated with retinoic acid as previously indicated. Prior to treatments, cells were serum starved for 4 hours. Following OGD, cultures were incubated in serum free D-MEM/F-12 media containing 5 mM N-methyl-D-aspartic acid (NMDA). After 6 hours, cultured media was replaced with 300 μL of warm PBS (37°C, pH 7.4) and background fluorescence was determined (*Fmin*). Cultures were then provided with 6 μM ethidium homodimer and incubated for 30 minutes at 37°C after which fluorescence was measured (*F*). Wells were then incubated with 0.03% saponin for 60 minutes at 37°C at which time a final measurement of fluorescence was taken (*Fmax*). Fluorescence data was collected using a Spectra Max Gemini M2 plate reader (Molecular Devices, Sunnyvale, CA, USA) at excitation/emission wavelengths of 530/620 with a cutoff of 610 nm. The percentage of dead cells was calculated using the following formula for each well:

(1) $$\%\;\text{Cell death}\;=\;\left(F\;x\;\text{Fmin}\right)/\left(\text{Fmax}\;-\;\text{Fmin}\right)\;x\;100$$

### Statistical Analysis

Indicated statistical tests were performed using GraphPad Prism Software (La Jolla, CA, USA).

## Abbreviations

ANOVA: Analysis of variance; CNS: Central nervous system; DHE: Dihydroethidium; DPI: Diphenylene iodonium; D-MEM/F-12: Dulbecco’s modified eagle medium/F-12; FCCP: Carbonyl cyanide 4-(trifluoromethoxy)phenylhydrazone; LTP: Long-term potentiation; I/R: Ischemia/reperfusion; NADPH Oxidase: Nicotinamide adenine dinucleotide phosphate-oxidase; NBT: Nitro blue tetrazolium chloride; NMDA: *N*-methyl-D-aspartic acid; NMDAR: *N*-methyl-D-aspartate receptor; NR2A/B: NMDA receptor subunit 2A/2B; OGD/R: Oxygen-glucose deprivation/reperfusion; PBS: Phosphate buffered saline; PSD-95: Post-synaptic density protein, 95 kDa; ROS: Reactive oxygen species; SFKs: Src family kinases.

## Competing interests

The authors declare that they have no competing interests.

## Authors’ contribution

PHB carried out the western blot analysis, immunoprecipitation, immunocytochemistry, NBT assay, viability assay, and draft of the manuscript. DAJ conceived of the study, participated in its design and coordination, performed the statistical analysis and helped to draft the manuscript. All authors read and approved the final manuscript.
